# Novel Polyvinyl Butyral/Monoacylglycerol Nanofibrous Membrane with Antifouling Activity

**DOI:** 10.3390/ma13173662

**Published:** 2020-08-19

**Authors:** Petra Peer, Jana Sedlaříková, Magda Janalíková, Liliana Kučerová, Pavel Pleva

**Affiliations:** 1 Institute of Hydrodynamics of the Czech Academy of Sciences, Pod Patankou 5/30, 16672 Prague, Czech Republic; peer@ih.cas.cz; 2 Department of Fat, Surfactant and Cosmetics Technology, Faculty of Technology, Tomas Bata University in Zlin, 275 Vavreckova, 76001 Zlin, Czech Republic; lili.zl@seznam.cz; 3 Department of Environmental Protection Engineering, Faculty of Technology, Tomas Bata University in Zlin, 275 Vavreckova, 76001 Zlin, Czech Republic; mjanalikova@utb.cz (M.J.); ppleva@utb.cz (P.P.)

**Keywords:** nanofibrous membranes, monoacylglycerols, polyvinyl butyral, wettability, antibacterial, antifouling activity

## Abstract

Monoacylglycerols (MAGs) have proven of great interest to the foodstuffs industry due to the promising antibacterial activity they show for controlling microbial contamination. Prior to this paper, this antibacterial agent had not been incorporated in a nanofibrous membrane. This study details convenient fabrication of nanofibrous membranes based on polyvinyl butyral (PVB) containing various concentrations of monocaprin (MAG 10) by an electrospinning process. Increasing the concentration of MAG 10 caused differences to appear in the shape of the nanofibers, in addition to which the level of wettability was heightened. Besides exhibiting antibacterial properties, the functional membranes demonstrated especially good antifouling activity. The novel and efficient nanofibrous membranes described have the potential to find eventual application in medical or environmental fields.

## 1. Introduction

The electrospinning technique allows the production of nanofibers from synthetic or natural polymers depending on the target or given application. Incorporating organic and inorganic materials in polymer solutions has recently become desirable in order to obtain novel electrospun composite nanofibers with additional properties, such as magnetic, antibacterial or antifouling activity, super-hydrophobicity, corrosion and chemical resistance [1,2,3]. Antibacterial nanofibrous membranes are flexible and highly porous, while their fibers possess a high surface-to-volume ratio and are ideal for various medical applications [4], food preservation [5], healthcare [6], protection textile fabrics [7] or water treatment [8]. Antibacterial agents are incorporated in the fibers by mixing them with the polymer solution prior to, during or after the electrospinning process, the latter via additional treatment. The most widely employed active agents in the formulation of nanofibers with antibacterial activity are silver or metal oxide nanoparticles, antibiotics, triclosan, quaternary ammonium salts, essential oils and chitosan [9,10,11,12]. Contemporary research on novel strategies seek to bring about new antimicrobial or antifouling substances that are safe and do not promote microbial resistance yet [13].

Monoacylglycerols (MAGs) are antibacterial agents commonly utilized by the pharmaceutical industry, as well as in the protection of textiles [14,15,16]. MAGs exhibit promising activity against Gram-positive and Gram-negative bacteria, yeasts and molds [17,18,19]. From the point of view of safety, mono- and di-acylglycerols of fatty acids have the status of GRAS (Generally Recognized as Safe) in the United States and within the European Union (EU) [20]. The antimicrobial properties of MAGs depend on the given molecular structure, i.e., the type and the length of a fatty acid carbon chain. They interfere with bacterial cell membranes and might cause cell lysis or a range of indirect effects which inhibit the cell metabolism [21]. Monocaprin, also known as glycerol monocaprate or 1-MAG of decanoic acid (MAG 10), has been found to reduce bacterial contamination effectively, thus enhancing safety and hygienic conditions [22]. Moreover, MAGs possess a specific amphiphilic structure, therefore, they are applicable as surfactants in the food industry, depending on their chain length. Several studies have shown the positive effects of surfactants on the character of electrospinning polymer solutions. Triton X-100 applied in poly (vinyl alcohol) solutions heightened homogeneity and prevented the formation of beads during the electrospinning process [23]. A similar effect was observed by Lin et al. [24] when Triton X-405 was added into a polystyrene solution.

Biofouling of polymeric membranes might be a major obstacle in the application of membrane-based technology for the purposes of water treatment. This phenomenon occurs through the colonization and growth of microorganisms, leading to the formation of biofilms on the membrane surfaces [25]. Materials benefiting from antifouling properties prevent the accumulation of biofilms on their surfaces either by repelling microorganisms or affecting the microbial biofilm structure (via steric repulsion or nanoscale rough topography), whereas antimicrobial coatings evince bacteriostatic or bactericidal activity by releasing an antimicrobial compound [26]. The most often surface-modified nanofibrous membranes applied in water treatment are prepared from polyvinylidene fluoride and its copolymers; nevertheless, the cellulose acetate or polyacrylonitrile are also widely used [27,28,29,30]. A comprehensive review on polymers for fabrication of nanofibrous membranes for water treatment is available in the literature [31].

Following prior experiments, polyvinyl butyral (PVB) was selected by the authors as the basis for preparing the polymer fibers detailed herein. This polymer is environment-friendly, non-toxic, odorless and it is a good candidate for fabrication at an industrial scale for its high productivity [32]. Polyvinyl butyral can be applied in various polymer blends, to enhance the spinning process of solutions, thus overcoming the problems of low solubility or rigid structure of some polymers [33]. It can even help to modify the fibers’ morphology; hence, it has been applied in the formulation of various functional fibers [33,34,35]. Fabricating PVB with Fe_2_O_3_ nanoparticles results in magnetic nanofibers, which have potential to be used in advanced membranes for effective removal of iron ions from groundwater [36]. The literature also reports on a PVB/CuO nanofiber-covered yarn with antibacterial properties, produced via a needle-less electrospinning system [37].

The aim of this study was to prepare PVB-based nanofibrous membranes containing MAG 10 by the electrospinning process for potential water treatment applications. The subsequent PVB/MAG 10 nanofibers were characterized by various methods, and the effect exerted by the concentration of MAG 10 on the final physicochemical and antibacterial properties was investigated.

## 2. Materials and Methods

### 2.1. Materials, Chemicals and Microorganisms

Polyvinyl butyral (*M*_w_ = 60,000 g mol^−1^; Mowital B 60T, kindly provided by Kuraray Specialities Europe, Troisdorf, Germany) was dissolved in ethanol (at the quality of p.a., Penta, Czech Republic). The structure of the Mowital B 60T comprised vinyl butyral, vinyl alcohol and vinyl acetate, at 75–81%, 24–27% and 1–4%, respectively. Decanoic acid, glycidol and chromium acetate hydroxide were supplied by Sigma-Aldrich (St. Louis, MO, USA). All the chemicals were applied as received without further purification. Both of the bacterial strains were obtained from the Czech Collection of Microorganism (CCM, Brno, Czech Republic), i.e., Gram-negative rods of *Escherichia coli* ATCC 25922 (CCM 3954), and Gram-positive cocci of *Staphylococcus aureus* ATCC 25923 (CCM 3953).

### 2.2. Preparation of 1-Monocaprin

1-monocaprin (MAG 10) was prepared proceeded by direct addition of decanoic acid into the glycidol by the epoxide ring opening, occurring in a double skin reactor at the temperature of 90 °C [38]. The product was then recrystallized from ethanol to the purity of 99%.

### 2.3. Preparation of Nanofibrous Membranes

Electrospinning was carried out to fabricate the nanofibrous membranes from various PVB solutions of various concentrations (8, 10 and 12 wt%) with different amounts of monocaprin (from 0.5 to 3 wt%). The lowest concentration point was based on the generally known antibacterial activity found in the literature [39]. The upper concentration limit followed the fact that higher than 3 wt% MAG 10 content led to the worse practical feasibility of the electrospinning process. A magnetic stirrer (Heidolph, Schwabach, Germany; set to 250 rpm at 25 °C) was used to homogenize the solutions (PVB/ethanol/MAG 10) over a period of 48 h. The nanofibrous webs were spun on a laboratory device (needle-less), which consisted of a high-voltage power supply (Spellman SL70PN150, Hauppauge, New York, NY, USA), a carbon steel stick (10 mm in diameter) and a motionless flat metal collector. Based on a previous study of the authors [40,41], neat PVB solutions were electrospun at 100 mm and at 20 kV. For the PVB/MAG 10 solutions, the conditions of the electrospinning process were modified, since the presence of the MAG 10 in the PVB solutions caused solvent evaporation to decelerate when drawing out the fibers. The electrospinning process was still carried out at a voltage of 20 kV, although the tip-to-collector distance was extended from 100 to 150 mm under ambient conditions (temperature 23 ± 1 °C, relative humidity 32% ± 1%). The drop of polymer solution placed on the tip measured approximately 0.2 mL.

### 2.4. Characterization of the Polymer Solutions

Rotational rheological measurements were performed on a Physica MCR 501 device (Anton Paar, Graz, Austria), equipped with concentric cylinders (26.6/28.9 mm inner/outer diameters) at a constant temperature of 25 °C. The shear rate applied when taking the rotational measurements ranged from 0.01 to 300 s^−1^. The viscosity measurement was done in triplicate. The conductivity of the PVB/MAGs solutions was gauged on a Conductivity Meter Lab 960 unit (SCHOTT Instruments, Mainz, Germany). Meanwhile, surface tension was observed via the Wilhelmy plate method (10 mm × 19.9 mm × 0.2 mm), using a Krüss K 100 force tensiometer (Krüss, GmbH, Hamburg, Germany). The conductivity and surface tension values are averages of five measurements.

### 2.5. Characterization of the Nanofibrous Membranes

Characterizing the morphology of the fibers required the use of a Vega 3 high-resolution scanning electron microscope (SEM) (Tescan, Brno, Czech Republic). Prior to imaging, a conductive layer was sputtered onto the samples. The mean diameters of the fibers were determined with the aid of Adobe Creative Suite software (CS5, Adobe Systems Inc., San Jose, CA, USA), wherein 300 fibers underwent analysis from 3 different images. The thickness of the nanofibrous layer was 21 ± 3 µm. The wettability of the nanofibrous membranes was measured by the sessile drop method at ambient temperature on a Theta optical tensiometer (Biolin Scientific, Västra Frölunda, Sweden) in combination with OneAttension software. Distilled water was applied as the reference liquid, the volume of droplet equaling 4 µL.

Further characterization of the prepared fibers was carried out by Fourier transform infrared spectroscopy (FTIR), with the Nicolet 6700 spectrometer (ThermoFisher Scientific, Waltham, MA, USA) having been set to attenuated total reflection (ATR) mode and fitted with a diamond crystal. Measurement conditions comprised 64 scans at the resolution of 2 cm^−1^ and the range of 4000–400 cm^−1^. Thermal analysis was performed by differential scanning calorimetry (DSC) on a Mettler Toledo DSC 700/1 device (Columbus, OH, USA) placed at the temperature range of −50 to 300 °C, and heating rate of 10 °C/min under a nitrogen atmosphere. The samples weighed approximately 5 mg. The results were evaluated from the first heating cycle of the samples. The measurements were done in triplicate.

### 2.6. Antibacterial Activity

The antibacterial properties of the electrospun nanofibers were evaluated from the samples of PVB (10 wt%) and PVB/MAG 10 (3 wt%) nanofibrous membranes by three different methods, described below: (a) agar disk diffusion method [42], (b) dynamic cultivation method, modified according to the literature [11,37] and (c) contact method in accordance with ISO 22196:2011 [43].

(a) The agar disk diffusion method encompassed the following: circular samples (9 mm in diameter) of nanofibers from neat PVB or PVB enriched with MAG 10 (3 wt%) on nonwoven textile were placed on agar plates previously inoculated with 1 mL of 0.5 McF turbid bacterial suspension (*Escherichia coli*, *Staphylococcus aureus*) in sterile saline solution. The plates were incubated at 37 °C for 24 h and the whole experiment was repeated three times. The authors evaluated the inhibition zones as well as growth under the samples.
(b) Samples of PVB or PVB/MAG 10 nanofibers on an aluminum foil substrate (disks of diameter 30 mm) were added into 100 mL flasks (Duran, DWK, Mainz, Germany) containing 25 mL Brain Heart Infusion (BHI) broth (Himedia Laboratories Pvt. Ltd., Mumbai, India) and bacteria (*Escherichia coli*, *Staphylococcus aureus*) at the concentration of 5 × 10^3^–5 × 10^4^ CFU/mL. The mixtures were shaken at 100 rpm on a shaking incubator at 37 °C. Then, the viable counts of bacteria present in the solution after the required contact time (60, 120, 300 and 1440 min) were determined via the spiral plate method on an Eddy Jet spiral plater (IUL, Barcelona, Spain). This experiment was performed in triplicate. The following Equation (1) was applied to calculate the rate of reduction in percent (% *R*):
  (1) $$\%\;R=\frac{\left(A-B\right)}{A}\cdot 100$$
  where *A* is the number of viable bacteria present in BHI with PVB nanofibers (control) after the given contact time, and *B* is the number of viable bacteria present in BHI with PVB/MAG 10 nanofibers (sample) after the same.
(c) Antibacterial activity was determined with adherence to the procedure stipulated under ISO 22196:2011 for *Escherichia coli* and *Staphylococcus aureus* [43]. Bacterial suspensions were prepared at concentrations ranging from 2.5 × 10^5^ to 1 × 10^6^ cells/mL. Four hundred µL of the suspension was deposited on a specimen 50 mm × 50 mm in size (neat PVB—Control untreated sample; PVB/MAG 10), which was covered with a square piece of polyethylene film (40 mm × 40 mm). The specimens and the films were placed under UV-light for 60 min to sterilize them prior to the experiments. Immediately (neat PVB), and after the contact time of 24 h, the specimens (neat PVB, PVB/MAG 10), both on aluminum foil, were rinsed with SCDLP broth (10 mL; soybean casein digest broth with lecithin and polyoxyethylene sorbitan monooleate) and the value for CFU/mL was determined. Log reduction in the number of living and viable cells of tested bacteria (*R*) was calculated according to Equation (2):
  (2) $$R=\left(U_{t}-U_{0}\right)-\left(A_{t}-U_{0}\right)$$
  where *U*_0_ is the average value for the common logarithm of the number of viable bacteria, in cells/cm^2^, recovered from the control samples (neat PVB nanofibers) immediately after inoculation, *U_t_* is the mean for the common logarithm of the number of viable bacteria, in cells/cm^2^, recovered from the control samples (neat PVB nanofibers) after 24 h and *A_t_* is the mean for the common logarithm of the number of viable bacteria, in cells/cm^2^, recovered from the test samples (PVB/MAG 10 nanofibers) after 24 h [43]. Reduction in the quantity of cells capable of growth by two orders of magnitude (*R* ≥ 2) was interpreted as a bactericidal effect of the tested nanofibers [44].

### 2.7. Antifouling Activity

It was intended to measure bacterial viable counts (Log CFU/mL) in planktonic form in comparison to attached (sessile) bacteria to nanofibrous membrane. The extent of antifouling activity was determined by a bacterial adhesion test modified according to Reference [45]. It was performed in 100 mL glass bottles (Duran, DWK, Mainz, Germany) that contained 25 mL of BHI broth (Himedia Laboratories Pvt. Ltd., Mumbai, India), the bacterial strain (*Escherichia coli* or *Staphylococcus aureus*) at the concentration of 10^4^–10^5^ CFU/mL and samples of the PVB nanofibrous membrane (neat 10 wt% PVB or 10 wt% PVB / 3 wt% MAG 10) on nonwoven circular disks (30 mm in diameter). The flasks were incubated at 37 °C for 24 h, then the membranes were taken out of the broth (a), washed, and immersed in sterile saline solution in conical tubes. The membranes were vigorously vortexed for 20 min to release attached bacteria from the nanofiber surfaces (b). The number of viable planktonic (a) and sessile (b) cells were determined by the spiral plate method described earlier (CFU/mL). This experiment was done in triplicate.

### 2.8. Statistical Analysis

Data were expressed as mean ± standard deviation (SD). Statistical analysis was carried out by a one-way analysis of variance (ANOVA) test in Statistica software (version 10, StatSoft, Inc., Tulsa, OK, USA) at the significance level of *p* < 0.05.

## 3. Results

### 3.1. Characterizatin of the Polymer Solutions

**Figure 1:**
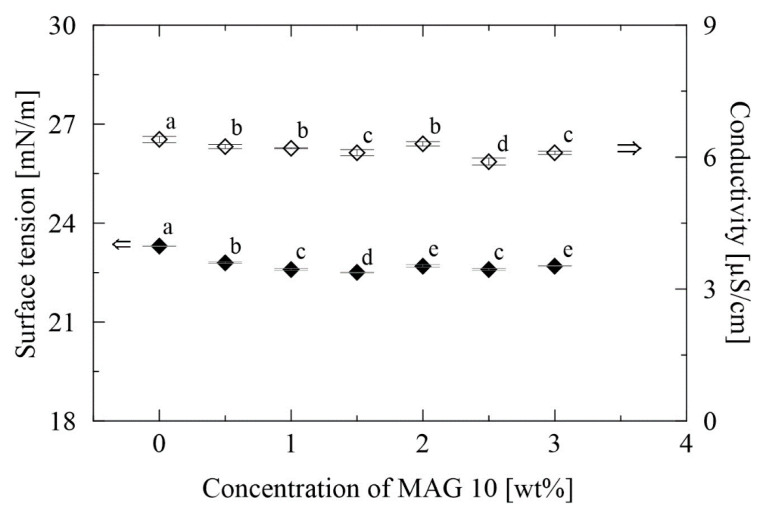
Surface tension and conductivity of the neat 10 wt% PVB and 10 wt% PVB/MAG 10 solutions. The various letters indicate significant differences (*p* < 0.05) between the samples.

The production of nanofibers and their resultant quality are affected by several parameters, such as electrical conductivity, viscosity and the surface tension of the polymer solution. Highly conductive solutions cause the formation of aggregated and fused fibers, although polymer solutions with high surface tension can avert this [46]. Three PVB solutions that varied in concentrations (8, 10 and 12 wt%) were prepared herein, into which different amounts of MAG were added. These exhibited surface tension and conductivity that did not appear to vary much in dependence on the amount of MAG 10 present (see Figure 1 and Supplementary Table S1), hence minimal impact seemed to be exerted on the formation of the fibers. The reason for this negligible effect on conductivity could have been that monoacylglycerols rank among non-ionic surfactants. A study by Juang et al. [47] reported similar findings, in that no significant impact on conductivity was observed when a non-ionic surfactant (nonylphenol) was added into the poly(vinyl alcohol) mixture. It is worth noting that for ionic surface active agents, electrical conductivity has been used to provide valuable data on interaction with a polymer [48].

The surface tension of the PVB solutions was measured by the Wilhelmy plate method at 25 ± 1 °C. Figure 1 shows that surface tension values of PVB samples supplemented with MAG 10 fluctuate around 22.9 mN/m. In spite of the results obtained by statistical analysis, these differences are not significant regarding the surface activity itself, compared with the surface tension of pure ethanol, which was 22.1 ± 0.05 mN/m. Even higher surface tension was observed in the PVB control samples (from 22.4 to 23.1 mN/m), with values slightly higher than in a previous study, where the mean of 21.9 mN/m was obtained [40]. Similarly, in the study of Reference [49], no substantial changes in surface tension were observed, when monoglyceride of lauric acid was incorporated into shellac polymer solution. The values for the surface tension of the modified PVB solutions ranged from 22.4 to 23.3 mN/m, revealing moderate differences between the concentrations of MAG. More significant findings (<0.05) were demonstrated in samples prepared from the various concentrations of PVB. However, all the surface tension values fluctuating around 22 mN/m indicate a quite high surface activity. This phenomenon can also be caused by the fact that applied MAG content exceeds its critical micelle concentration that is crucial for the surface activity of each surfactant. The complete results of conductivity and surface tension of all the samples are included in the Supplementary Material as Table S1.

**Figure 2:**
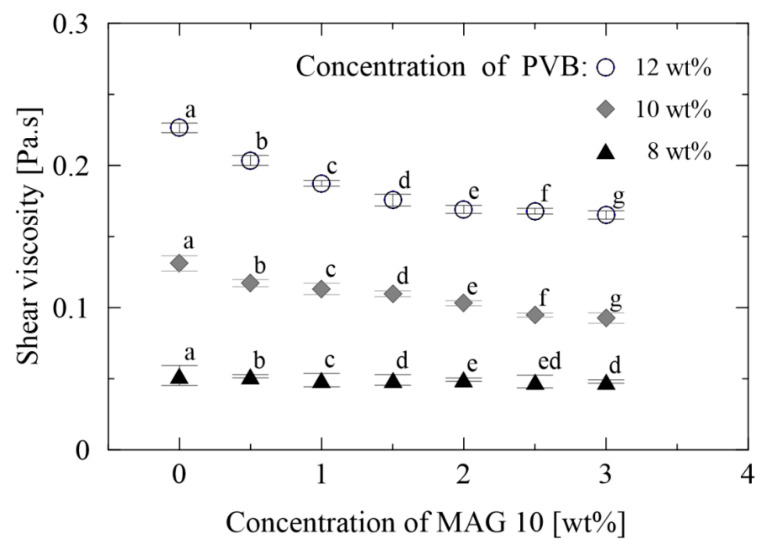
Shear viscosity of the neat PVB and PVB/MAG 10 solutions. The various letters indicate significant differences (*p* < 0.05) between the samples.

The rheological parameters of the polymer solutions are influenced by the molecular weight of the polymer, its concentration and the nature of the solvents. In this case, the molecular weight and type of solvent were fixed, while the effect exerted by concentration was investigated. The neat PVB solutions demonstrated Newtonian behavior, concurring with a previous study by the authors [40,50]. The presence of MAG 10 in the PVB solutions did not engender Newtonian behavior by these complex fluids. Figure 2 reveals that the viscosities of all three PVB/MAG 10 solutions decreased alongside an increase in the amount of MAG 10, a phenomenon potentially associated with an emulsifying effect of the surfactant. Shear viscosity remained more or less constant, nevertheless, from around the point MAG 10 amounted to 1.5 wt%. This behavior was predictable since no cooperative binding had been expected between the PVB chain and non-ionic monoacylglycerol [51].

### 3.2. Characterization of Nanofibrous Membranes

**Figure 3:**
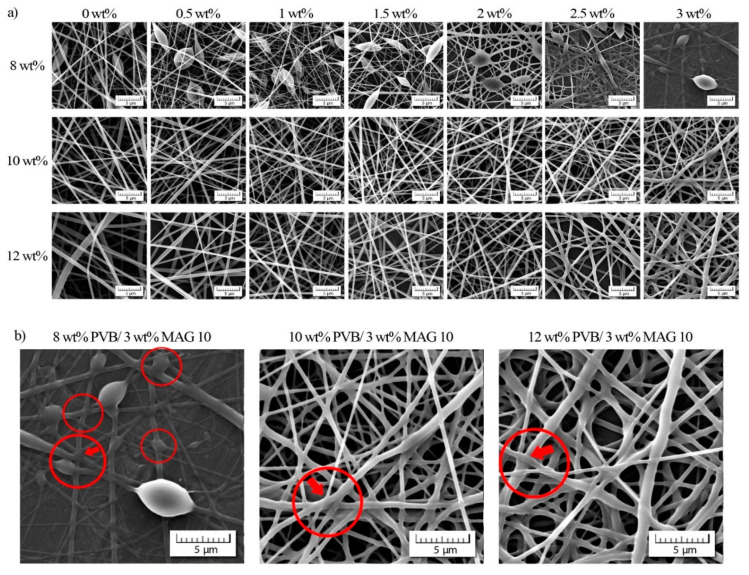
(**a**) Scanning electron microscope (SEM) images of the neat PVB and PVB/MAG 10 solutions, and (**b**) detail of fiber deformation.

A study was made as to the effect of PVB and MAG 10 concentration on morphological properties. SEM images for all the nanofibrous membranes prepared are displayed in Figure 3a, detailing beaded and flat, uniform fibers in dependence on the concentration of PVB applied. Increasing the concentration of monocaprin caused the resulting fibers to change in shape, going through the minimum fiber diameter up to 1 to 1.5 wt%, followed by a renewed increase. The fused fibers’ structure is shown in case of the lowest PVB concentration (8 wt%), while raising the concentration of PVB suppressed this negative trend in shape. The structural changes from uniform to fused were probably caused by the mutual miscibility of PVB matrix and monocaprin, which led to incorporation of MAG 10 into the inner structure followed by deformation of fibers (see Figure 3b).

**Figure 4:**
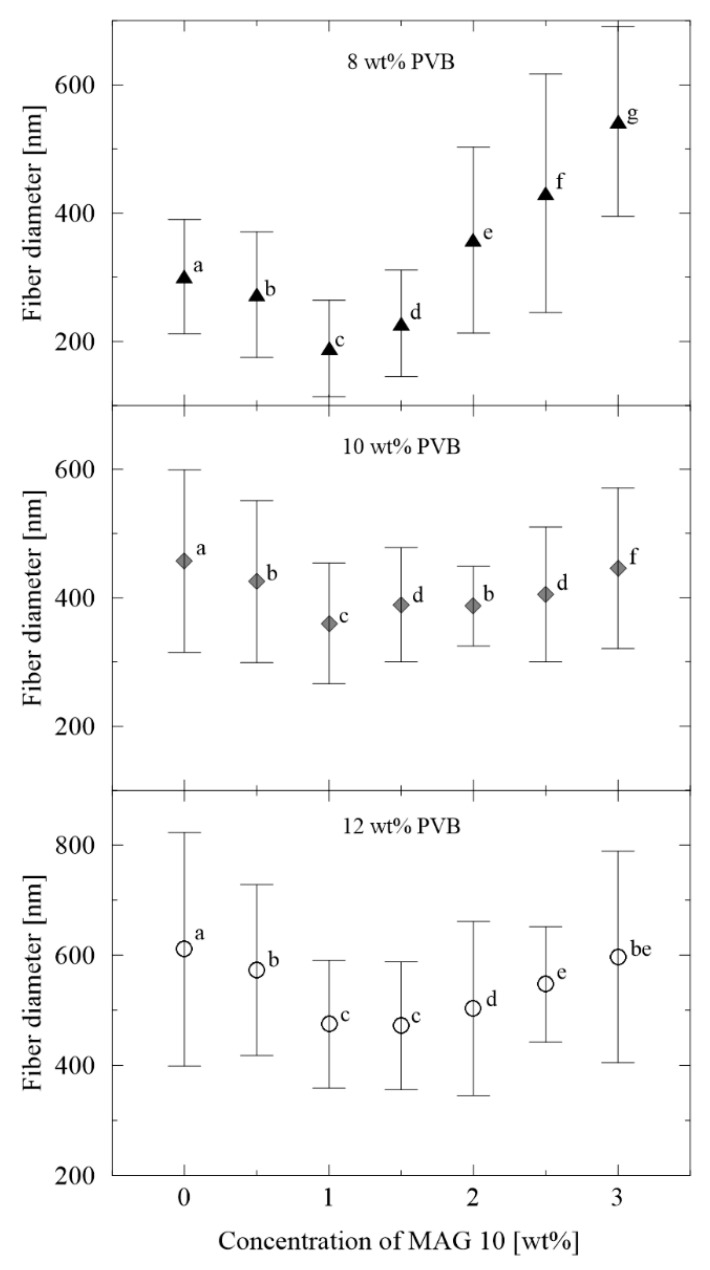
Fiber diameter vs. concentration of MAG 10. The various letters indicate significant differences (*p* < 0.05) between the samples.

Mean averages for fiber diameter were calculated from 300 individual fibers for each sample, as plotted in Figure 4. The diameters of the neat PVB nanofibers increased from 300 to 600 nm in parallel with the rise in concentration from 8 to 12 wt%, which is consistent with the literature [52]. Contrary to this was the finding that heightening the amount of MAG 10 initially led to a decrease in the diameters of the nanofibers, a phenomenon which ceased from the concentration of 1 wt%, when they thickened. A similar trend was observed in a study by Abutaleb et al., who investigated polyetherimide-based fibers enriched with different ionic and non-ionic surface active agents [53].

**Figure 5:**
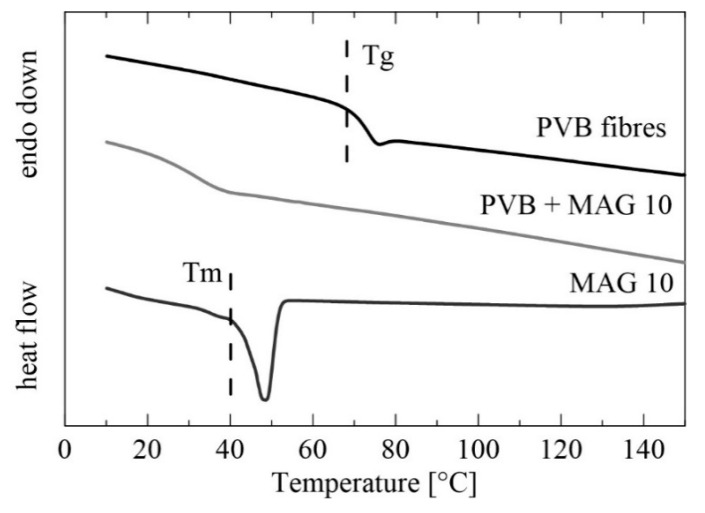
Differential scanning calorimetry (DSC) thermograms of MAG 10, the PVB fibers and PVB fibers with MAG 10 (first heating scan).

Figure 5 presents DSC patterns for the PVB nanofibers from the first heating scan, for which the temperature range of 0–150 °C was selected. In the case of the unmodified PVB fibers (12 wt%), a glass transition temperature (Tg) was observed at 61 °C, comparable with data obtained for a PVB membrane prepared by solvent casting reported in Reference [54]. Adding monocaprin resulted in suppressing the T_g_ peak and shifting it to a lower value, ca 40 °C, and this was possibly caused by partial miscibility of the PVB matrix and MAG 10 [55]. This statement can be supported even by the melting temperature determination. Whereas, a thermogram of MAG 10 shows one sharp endothermic peak of approximately 48 °C corresponding to the melting temperature (T_m_) of monoacylglycerol, no melting peak was observed in the PVB/MAG 10 sample. Similar results obtained by DSC characterization were proved in the study of Vasoya et al. [56], in which various polyglycerides have been investigated as potential carriers for poorly water-soluble drugs. Miscibility of PVB fibers and MAG 10 can be also presumed based on the SEM results (Figure 3), where a change in fibers’ shape was observed with increasing monocaprin concentration.

**Figure 6:**
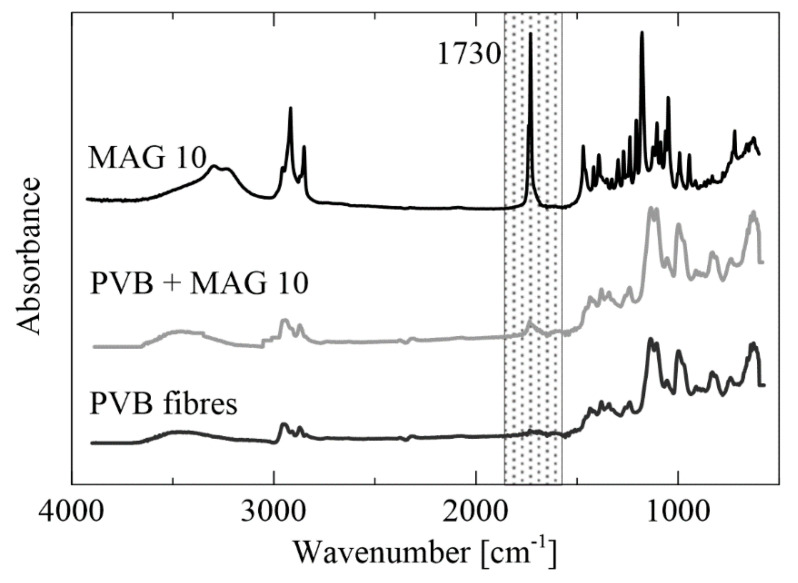
Fourier transform infrared spectroscopy (FTIR)-ATR spectra of the neat PVB and PVB modified with MAG 10.

The results from FTIR analysis of the PVB fibers (12 wt%) and PVB enriched with MAG 10 (3 wt%) are given in Figure 6. Spectra of MAG 10 showed peaks at 2950 cm^−1^, 2850 cm^−1^ and 1730 cm^−1^, indicating carboxylic acid bands. The PVB sample showed characteristic peaks for this polymer, with vibrations at 1130 and 997 cm^−1^ corresponding to the C–O–C bonds of the cyclic acetal group. The broad peak around 3450 cm^−1^ indicates hydroxyl bonds [54]. In PVB fibers supplemented with MAG 10, the PVB groups were preserved and a new peak at 1730 cm^−1^ was shown, corresponding to C=O carbonyl stretch.

**Figure 7:**
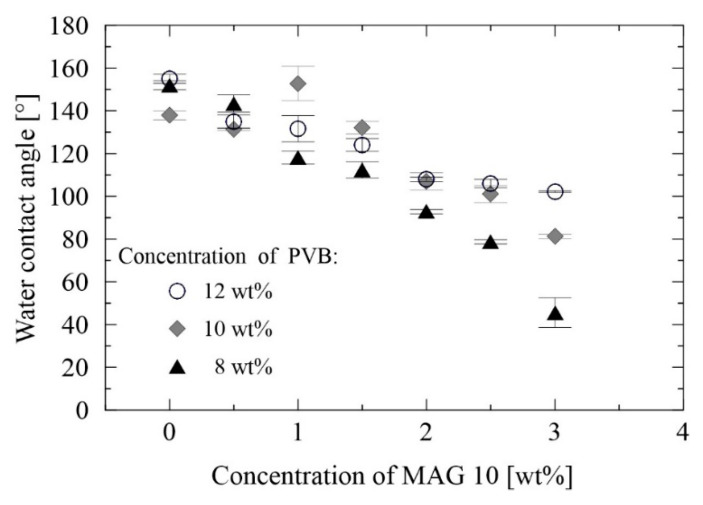
Wettability of the neat PVB and PVB modified with MAG 10.

**Figure 8:**
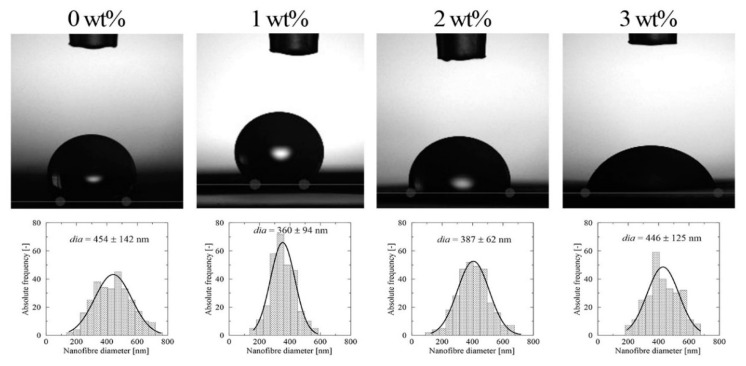
Fiber distribution and wettability of the neat 10 wt% PVB and 10 wt% PVB with MAG 10 membranes.

The surface properties of membranes play a critical role in their practical applicability. It is known that hydrophilic surfaces with minimum contact angles exhibit lower capillary pressure of the filter media, thus enhancing the flow rate of water molecules through the membrane. The tendency for fouling diminishes when there is a greater number of hydrophilic membranes present, as weaker interactions occur between the organic molecules and polar membranous substrate [57]. In order to study the wettability of the PVB samples and the effect exerted by modification with MAG 10, static water contact angles (WCA) were measured by the sessile drop method. The PVB membranes generally proved to be strongly hydrophobic, for which contact angles from 137° to 155° were observed (Figure 7), in agreement with the literature [58]. It is obvious that incorporating MAG 10 caused a drop in the contact angles for all the tested PVB concentrations, probably due to the polar group terminated molecule. The lowest contact angle (45°) was monitored in the sample containing 8 wt% PVB and 3 wt% MAG 10. Wettability is additionally affected by the morphology of fibrous membranes. As shown in Figure 8, the morphology of fibers correlated with the WCA of 10 wt% PVB containing various concentrations of MAG 10. These findings concur with the SEM analysis, where a non-homogeneous and enlarged fiber structure was observed, potentially bringing about heightened wettability. Such enhancement of wetting behavior could have been engendered through migration of the polar groups of the surfactant to the surface of the nanofibers during solidification [59]. Following this, strong hydrogen bond interactions between the surfactant and water molecules are expected. Besides the type of active substance used, the method of fibers’ preparation is important [60].

### 3.3. Antibacterial Efficiency

#### 3.3.1. Disk Diffusion Method

The antibacterial activity of the PVB and PVB/MAG 10 nanofibrous membranes was gauged by the agar disk diffusion method. MAGs have previously been reported to be effective against various Gram-negative and Gram-positive bacteria [61]. By this method, no inhibition zones on the agar plates against *E. coli* and *S. aureus* were observed in this work testing PVB and PVB/MAG 10 nanofibrous membranes. It can be supposed that the MAG 10 was probably not able to release from nanofibers at high concentration, and hence could not diffuse and prevent microbial growth in zones. The only observed difference was that both bacterial strains grew under the PVB nanofibrous membranes, however no bacterial growth was monitored on the agar medium below the PVB/MAG 10 nanofibrous membranes. These results support an idea that the monocaprin had been successfully embedded in the nanofibers and potentially diffused at very low concentrations. This phenomenon was also observed by Park et al. [45], who tested PVA nanofibers supplemented with benzyltriethyl ammonium chloride as an antimicrobial agent.

#### 3.3.2. Dynamic Cultivation Method

In order to verify the antibacterial activity in broth during cultivation, a dynamic cultivation method was performed. Reduction in the viable bacterial counts (*E. coli* and *S. aureus*) was evaluated in tubes with the PVB/MAG 10 nanofibers after 60, 120, 300 and 1440 min, and these findings were subsequently compared to those for neat PVB. As shown in Table 1, the PVB/MAG 10 membrane exhibited no effect against *E. coli* at any time. A slightly different trend was reported by Thormar et al. [62], though, who investigated the emulsion with monocaprin (5 mM), therein showing a single log for *E. coli* in comparable pH. It is obvious that the type of carrier matrix of monocaprin is crucial as to the resultant antibacterial effect. Nevertheless, the concentration of released MAG 10 is undoubtedly lower and is not effective against the *E. coli* cells. *S. aureus* demonstrated an approximate 20% reduction in log when in contact with the PVB/MAG 10 nanofibrous membrane for up to 300 min in shaken broth. Nevertheless, no reduction in log was noticed after 1440 min. Thus, PVB/MAG 10 nanofibers in the cultivation broth prolonged the adaptation phase of the growth curve for *S. aureus*. These results agreed with those of the previous studies [18,39], where 1-monoacylglycerols were observed to be more efficient against Gram-positive strains as a consequence of the different structure of their cell walls. Monocaprin in a microemulsion has also proved to exert a greater antibacterial effect against Gram-positive *Micrococcus luteus* and *Bacillus cereus* than Gram-negative bacteria [15]. In brief, it was demonstrated that MAG 10 could be released from the nanofibrous membranes into the broth at very low concentrations. Although insufficient to kill bacteria, it had the capability to slow down the growth of *S. aureus*.

#### 3.3.3. Contact Method

The antibacterial activity of the PVB and PVB/MAG 10 nanofibrous membranes was checked against *Staphylococcus aureus* and *Escherichia coli* by the contact method, in accordance with ISO 22196:2011. The numbers of viable bacteria recovered were determined and the tests were deemed valid according ISO 22196:2011 conditions. The resulting efficacy is given as a log reduction against a control, with minimum log 2 reduction being required to pass the test specification [63]. The results of this experiment indicated that bacterial growth on the PVB/MAG 10 nanofibers was reduced by more than 2 or 3 logs, hence they were in compliance [64]. The reduction in viable cell numbers in this study was calculated as *R* = 3.8 for *S. aureus* and *R* = 2.3 for *E. coli*, again revealing a higher efficiency against Gram-positive bacteria. Since the nanofibrous membrane of the PVB enriched with MAG 10 had not been investigated before, no comparable findings were available in the literature. As regards other systems, comparable data were acquired from a source [64] wherein a polyurethane membrane with 0.04 mg/cm^2^ Ag/ZnO microparticles had a similar antibacterial effect against *S. aureus* and *E. coli*, albeit at higher Ag/ZnO concentrations. Such activity also rose to *R* > 6.7 for *E. coli* and *R* > 4.8 for *S. aureus*.

### 3.4. Antifouling Activity

The antifouling activity of the PVB nanofibrous membranes was determined by a bacterial adhesion test. An initial bacterial attachment is mediated by electrokinetic and hydrophobic interactions [65]. Hydrophilicity is thus a desired property of a filtration membrane that has the ability to heighten permeation and engender an antifouling effect [66]. Bacterial viable counts in planktonic form (Log CFU/mL) in comparison to attached (sessile) bacteria to nanofibrous membrane were measured in liquid medium with PVB or PVB/MAG 10 nanofiber membranes. It is worthy of note that the sessile cells are attached to the nanofibrous membrane, and this could lead to formation of a biofilm layer [65].

**Figure 9:**
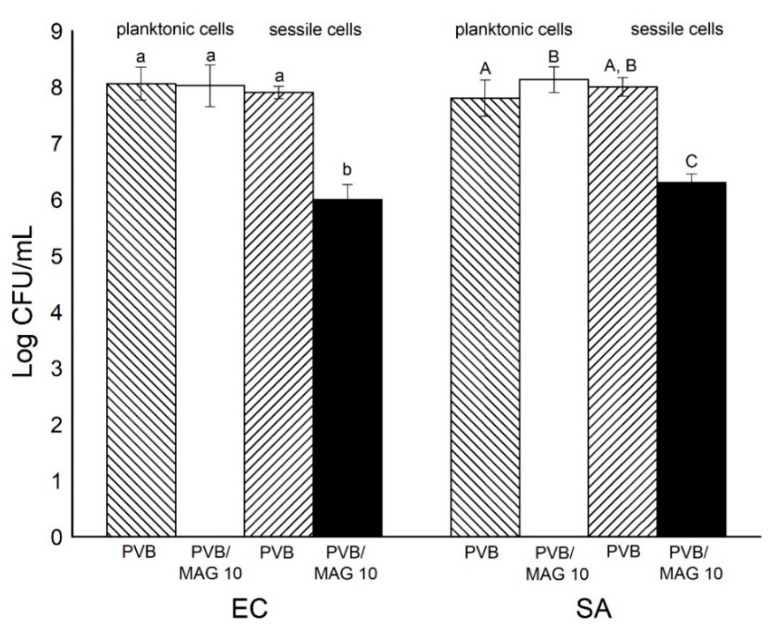
Bacterial adhesion test (antifouling activity): the numbers of viable bacteria *Escherichia coli* (EC) and *Staphylococcus aureus* (SA) in planktonic and sessile mode determined after the contact time (24 h) with the PVB or PVB/MAG 10 membranes. The various lowercase and uppercase letters indicate significant differences (*p* < 0.05) between the samples tested against EC and SA, respectively.

In accordance with the results described above (Section 3.3), also, no reduction effect of PVB/MAG 10 nanofibrous membranes to planktonic bacteria was noticed (Figure 9). Surprisingly, a significant difference was found in sessile cell counts attached to PVB/MAG 10 nanofiber membranes in comparison with the control PVB membrane without monocaprin (Figure 9). It can be explained that adding the monocaprin caused a rise in hydrophilicity. An extent of 2 log reductions in *E. coli* sessile cells and almost the same result for *S. aureus* was obtained.

Anti-infective surfaces are classified as antimicrobial or antifouling, based upon their different mode of action against microorganisms—the first being able to kill microbes when they approach the surfaces, the other preventing microbial accumulation either via repellent properties against microorganisms or interference with the structure of a biofilm [26]. In summary, the PVB/MAG 10 nanofibrous membranes detailed herein, in light of their properties and subsequent results, are capable of interesting antifouling activity (see Figures S1 and S2 in Supplementary Materials) and can potentially prevent the biofilm formation.

To sum up, the PVB/MAG 10 nanofibrous membranes were seen to exert an antibacterial effect when in direct contact with the bacteria (according to contact method, Section 3.3.3), although an insufficient concentration of MAG 10 was released into the water environment (dynamic cultivation method, Section 3.3.2), thus little bactericidal or bacteriostatic effect was observed against *E. coli* and *S. aureus*. In both methods, PVB/MAG 10 nanofibrous membrane was more efficient against *S. aureus*. On the other hand, the same antifouling effect was observed with both bacterial strains, which supports an idea that this effect is caused by physical and mechanical properties of PVB/MAG 10 nanofibers. Following the obtained results, further research will focus on the investigation of some other factors, such as the molecular structure of applied active agent (chain length) and mutual interactions with the polymer to get more complex information on nanofibrous membranes as regards their antifouling activity, as well as the conditions of potential release.

## 4. Conclusions

Novel antibacterial nanofibrous membranes were successfully prepared by electrospinning PVB/MAG 10 polymer solutions. The fabricated membranes exhibited a reduction in water contact angle alongside a rise in concentration of monocaprin in the PVB. The diameters of the neat PVB nanofibers proportionally increased in parallel with higher PVB concentration. The PVB/MAG 10 fibers showed initial diminishment in diameter, after which an increase was observed, depending on the content of MAG 10. Fiber deformation was observed, due to monocaprin embedded in the inner structure. Antibacterial tests proved that the monocaprin incorporated in the nanofibrous membranes significantly inhibited the growth of *S. aureus* bacteria, whereas lesser activity was observed against *E. coli* when in direct contact. Moreover, due to its anti-infective surface, the PVB/MAG 10 nanofibrous membrane exhibited great antifouling activity in comparison with the neat PVB nanofibers. For these reasons, the functionalized PVB/MAG 10 system shows potential as an antifouling filtration membrane for the purposes of wastewater treatment.

## Figures and Tables

**Table 1:** Percent reduction (% *R*) of *Escherichia coli* and *Staphylococcus aureus* at intervals of contact with the PVB/MAG 10 nanofibrous membrane related to neat PVB.

	*Escherichia coli* ATCC 25922	*Staphylococcus aureus* ATCC 25923
Time (min)	% R	% R
60	−1.61 ^a^	22.83 ^a^
120	−0.72 ^a^	18.76 ^a^
300	−7.76 ^a^	22.12 ^a^
1440	−7.49 ^a^	−5.72 ^b^

^a,b^: Different letters in the same column indicate significant differences (*p* < 0.05).

## Supplementary Materials

The following are available online at https://www.mdpi.com/1996-1944/13/17/3662/s1, Table S1: Dependence of surface tension and electrical conductivity on the MAG 10 concentration in the all PVB solutions. Figure S1: Fluoresence images of the (a) PVB and (b) PVB/MAG 10 sample after 24 h cultivation *(Escherichia coli)*. Figure S2: Fluorescence images of the (a) PVB and (b) PVB/MAG 10 sample after 24 h cultivation (*Staphylococcus aureus*).
