# Supplementary material for: Novel Polyvinyl Butyral/Monoacylglycerol Nanofibrous Membrane with Antifouling Activity
Source: Materials (Basel). 2020 Aug 19;13(17):3662. doi: 10.3390/ma13173662 (PMC7504434; doi:10.3390/ma13173662)
Supplement: Supplementary file 1 [file materials-13-03662-s001.pdf]

materials

Supplementary Materials: Novel Polyvinyl Butyral/Monoacylglycerol Nanofibrous Membrane with Antifouling Activity

Petra Peer [1], Jana Sedlaříková [2,*], Magda Janalíková [3], Liliana Kučerová [2] and Pavel Pleva [3]

1 Institute of Hydrodynamics of the Czech Academy of Sciences, 16672 Prague, Czech Republic; peer@ih.cas.cz
2 Department of Fat, Surfactant and Cosmetics Technology, Faculty of Technology, Tomas Bata University in Zlin, 275 Vavreckova, 76001 Zlin, Czech Republic; lili.zl@seznam.cz
3 Department of Environmental Protection Engineering, Faculty of Technology, Tomas Bata University in Zlin, 275 Vavreckova, 76001 Zlin, Czech Republic; mjanalikova@utb.cz (M.J.); ppleva@utb.cz (P.P.)
* Correspondence: sedlarikova@utb.cz

Table S1. Dependence of surface tension and electrical conductivity on the MAG 10 concentration in the all PVB solutions.

	Surface tension (mN/m)			Conductivity (uS/cm)		
**MAG (wt%)**	**8% PVB**	**10% PVB**	**12% PVB**	**8% PVB**	**10% PVB**	**12% PVB**
**0**	$22.4{\pm}0.02^{aA}$	$23.3{\pm}0.12^{aB}$	$23.1{\pm}0.09^{aC}$	$5.7{\pm}0.04^{aA}$	$6.4{\pm}0.07^{aB}$	$5.0{\pm}0.08^{aC}$
**0.5**	$22.4{\pm}0.01^{aA}$	$22.8{\pm}0.06^{bB}$	$23.0{\pm}0.02^{aC}$	$6.3{\pm}0.08^{bA}$	$6.2{\pm}0.05^{bA}$	$6.6{\pm}0.56^{bA}$
**1**	$22.2{\pm}0.03^{bA}$	$22.6{\pm}0.03^{cB}$	$23.1{\pm}0.08^{aC}$	$6.0{\pm}0.04^{cA}$	$6.2{\pm}0.11^{bB}$	$6.0{\pm}0.07^{bA}$
**1.5**	$22.4{\pm}0.02^{aA}$	$22.5{\pm}0.01^{dB}$	$22.8{\pm}0.02^{bC}$	$6.0{\pm}0.05^{cA}$	$6.1{\pm}0.07^{cA}$	$5.1{\pm}0.07^{cB}$
**2**	$22.6{\pm}0.03^{cA}$	$22.7{\pm}0.04^{eB}$	$23.0{\pm}0.01^{aC}$	$6.0{\pm}0.11^{cA}$	$6.3{\pm}0.05^{bB}$	$5.6{\pm}0.08^{dC}$
**2.5**	$22.4{\pm}0.01^{aA}$	$22.6{\pm}0.03^{cB}$	$23.0{\pm}0.02^{aC}$	$5.8{\pm}0.04^{dA}$	$5.9{\pm}0.08^{dA}$	$5.0{\pm}0.09^{aB}$
**3**	$22.6{\pm}0.04^{cA}$	$22.7{\pm}0.01^{eB}$	$23.0{\pm}0.04^{aC}$	$5.7{\pm}0.04^{aA}$	$6.1{\pm}0.04^{cB}$	$5.4{\pm}0.00^{eC}$

$^{a\text{–}d}$ and $^{A\text{–}C}$: different lower-case/upper-case letters in the same column/line indicate significant differences, respectively ($p < 0.05$).

Fluorescence microscopy:

Fluorescence microscopy was performed using a fluorescence microscope (Olympus BX53, 200x magnification, Hamburg, Germany) equipped with Microscope Digital Camera DP73, Olympus (Tokyo, Japan) evaluated by the cellSens Standard 1.18 Olympus (Tokyo, Japan) software. Analysis was carried out on minimum 20 positions in three replicates. Preparation of samples was carried out by the help of LIVE/DEAD™ BacLight™ Bacterial Viability Kit (Thermo Fischer, USA), based on the protocol [1] with slight modifications.

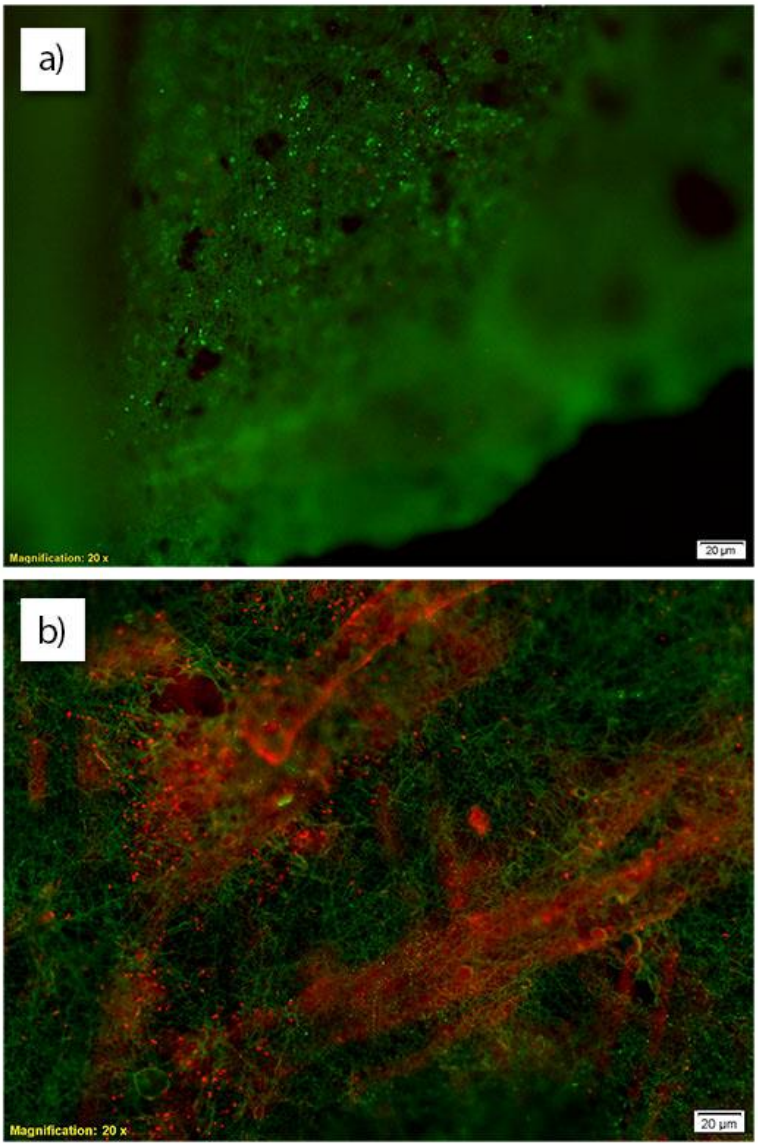

Figure S1. Fluoresence images of the **a**) PVB and **b**) PVB/MAG 10 sample after 24h cultivation *(Escherichia coli)*.

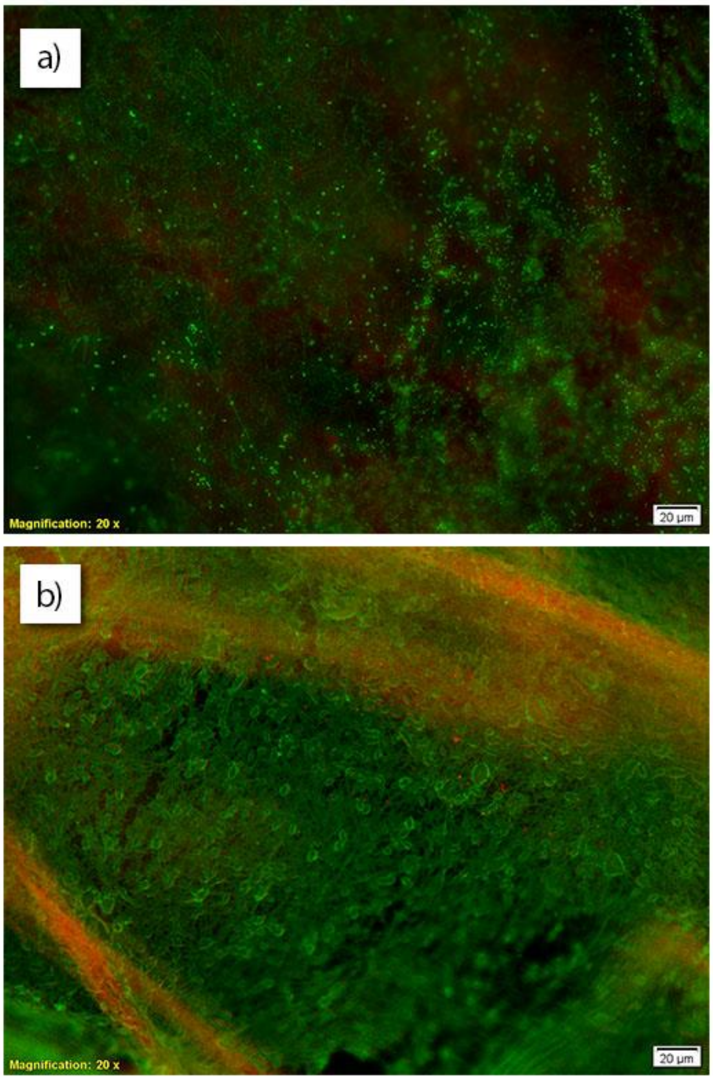

Figure S2. Fluorescence images of the **a**) PVB and **b**) PVB/MAG 10 sample after 24h cultivation (*Staphylococcus aureus*).

Reference

1. Molecular Probes, Invitrogen detection technologies [online]. Molecular Probes, Inc. Available at: https://assets.thermofisher.com/TFS-Assets/LSG/manuals/mp07007.pdf.

© 2020 by the authors. Submitted for possible open access publication under the terms and conditions of the Creative Commons Attribution (CC BY) license (http://creativecommons.org/licenses/by/4.0/).
